# Associations of Early-Life Exposure to Submicron Particulate Matter With Childhood Asthma and Wheeze in China

**DOI:** 10.1001/jamanetworkopen.2022.36003

**Published:** 2022-10-11

**Authors:** Chuansha Wu, Yunquan Zhang, Jing Wei, Zhuohui Zhao, Dan Norbäck, Xin Zhang, Chan Lu, Wei Yu, Tingting Wang, Xiaohong Zheng, Ling Zhang

**Affiliations:** 1 Department of Environmental Hygiene and Occupational Medicine, School of Public Health, Medical College, Wuhan University of Science and Technology, Wuhan, China; 2 Hubei Province Key Laboratory of Occupational Hazard Identification and Control, Wuhan University of Science and Technology, Wuhan, China; 3 Department of Epidemiology and Biostatistics, School of Public Health, Medical College, Wuhan University of Science and Technology, Wuhan, China; 4 Department of Chemical and Biochemical Engineering, Iowa Technology Institute, The University of Iowa, Iowa City; 5 Department of Environmental Health, School of Public Health, Fudan University, Shanghai, China; 6 Department of Medical Sciences, Uppsala University, Uppsala, Sweden; 7 Research Centre for Environmental Science and Engineering, Shanxi University, Taiyuan, China; 8 Department of Occupational and Environmental Health, School of Public Health, Xiangya Medical College, Central South University, Changsha, China; 9 Joint International Research Laboratory of Green Buildings and Built Environments, Ministry of Education, Chongqing University, Chongqing, China; 10 School of Nursing and Health Management, Shanghai University of Medicine and Health Sciences, Shanghai, China; 11 School of Energy and Environment, Southeast University, Nanjing, China

## Abstract

**Importance:**

Exposure to particulate matter (PM) has been associated with childhood asthma and wheeze. However, the specific associations between asthma and PM with an aerodynamic equivalent diameter of 1 μm or less (ie, PM_1_), which is a contributor to PM_2.5_ and potentially more toxic than PM_2.5_, remain unclear.

**Objective:**

To investigate the association of early-life (prenatal and first year) exposure to size-segregated PM, including PM_1_, PM_1-2.5_, PM_2.5_, PM_2.5-10_, and PM_10_, with childhood asthma and wheeze.

**Design, Setting, and Participants:**

This cross-sectional study was based on a questionnaire administered between June 2019 and June 2020 to caregivers of children aged 3 to 6 years in 7 Chinese cities (Wuhan, Changsha, Taiyuan, Nanjing, Shanghai, Chongqing, and Urumqi) as the second phase of the China, Children, Homes, Health study.

**Exposures:**

Exposure to PM_1_, PM_1-2.5_, PM_2.5_, PM_2.5-10_, and PM_10_ during the prenatal period and first year of life.

**Main Outcomes and Measures:**

The main outcomes were caregiver-reported childhood asthma and wheeze. A machine learning–based space-time model was applied to estimate early-life PM_1_, PM_2.5_, and PM_10_ exposure at 1 × 1-km resolution. Concentrations of PM_1-2.5_ and PM_2.5-10_ were calculated by subtracting PM_1_ from PM_2.5_ and PM_2.5_ from PM_10_, respectively. Multilevel (city and child) logistic regression models were applied to assess associations.

**Results:**

Of 29 418 children whose caregivers completed the survey (15 320 boys [52.1%]; mean [SD] age, 4.9 [0.9] years), 2524 (8.6%) ever had wheeze and 1161 (3.9%) were diagnosed with asthma. Among all children, 18 514 (62.9%) were breastfed for more than 6 months and 787 (2.7%) had parental history of atopy. A total of 22 250 children (75.6%) had a mother with an educational level of university or above. Of the 25 422 children for whom information about cigarette smoking exposure was collected, 576 (2.3%) had a mother who was a current or former smoker during pregnancy and 7525 (29.7%) had passive household cigarette smoke exposure in early life. Early-life PM_1_, PM_2.5_, and PM_10_ exposure were significantly associated with increased risk of childhood asthma, with higher estimates per 10-μg/m^3^ increase in PM_1_ (OR, 1.55; 95% CI, 1.27-1.89) than in PM_2.5_ (OR, 1.14; 95% CI, 1.03-1.26) and PM_10_ (OR, 1.11; 95% CI, 1.02-1.20). No association was observed between asthma and PM_1-2.5_ exposure, suggesting that PM_1_ rather than PM_1-2.5_ contributed to the association between PM_2.5_ and childhood asthma. There were significant associations between childhood wheeze and early-life PM_1_ exposure (OR, 1.23; 95% CI, 1.07-1.41) and PM_2.5_ exposure (OR, 1.08; 95% CI, 1.01-1.16) per 10-μg/m^3^ increase in PM_1_ and PM_2.5_, respectively.

**Conclusions and Relevance:**

In this cross-sectional study, higher estimates were observed for the association between PM with smaller particles, such as PM_1_, vs PM with larger particles and childhood asthma. The results suggest that the association between PM_2.5_ and childhood asthma was mainly attributable to PM_1_.

## Key Points

**Question:**

Is early-life exposure to particulate matter (PM), especially with an aerodynamic equivalent diameter of 1 μm or less (ie, PM_1_), associated with increased risk of childhood asthma?

**Findings:**

In this cross-sectional study of 29 418 Chinese children aged 3 to 6 years, early-life PM_1_, PM_2.5_, and PM_10_ exposure was associated with increased risk of childhood asthma, with higher estimates for smaller particles. Moreover, PM_1_ rather than PM_1-2.5_ contributed to the association between PM_2.5_ and asthma.

**Meaning:**

The findings suggest that PM with smaller particles may be more toxic to the respiratory system than PM with larger particles; health care policy makers should pay more attention to early-life PM_1_ exposure to reduce childhood asthma associated with PM.

## Teaser

This cross-sectional study examines the association of early-life exposure to particulate matter of varying particle sizes with asthma and wheeze among children aged 3 to 6 years in 7 cities in China.

## Introduction

Ambient particulate matter (PM) pollution has aroused great interest and attention from all over the world because of its association with a substantial global burden of disease.^1,2,3,4^ Although air quality in China has improved in recent years, PM exposure levels were still associated with approximately 1.4 million deaths in China in 2019.^5^ Recent epidemiological studies indicated that long-term and short-term PM exposure have positive associations with respiratory disease in vulnerable populations, such as children.^6,7^

Asthma is the most common chronic respiratory disease in children, with a trend of increasing prevalence.^8,9,10,11^ The increase in prevalence of asthma may be associated with environmental factors independently or combined with genetic factors.^12,13,14,15^

Previous studies estimated that PM_1_ (PM with an aerodynamic equivalent diameter ≤1 μm) is a major contributor (approximately 80%) to the concentration of PM_2.5_ (PM with an aerodynamic equivalent diameter ≤2.5 μm) in China.^16,17,18^ Emerging evidence indicated that the particle size of PM was inversely associated with lung toxic effects.^19,20^ To our knowledge, only 2 epidemiological studies have reported a positive association between PM_1_ exposure and childhood asthma, perhaps because PM_1_ is not routinely monitored worldwide. One of these studies estimated PM_1_ exposure at 10 × 10-km resolution,^21^ which may be subject to exposure misclassifications. The other was a single-city study,^22^ although the vast territory of China makes it hard to generalize results from a single city to the whole country. Moreover, previous studies did not elucidate whether the associations between PM_2.5_ and asthma were mainly owed to the contribution of PM_1_. Within a multicity population, we intended to investigate the association of exposure to 1 × 1-km high-resolution, size-segregated PM (PM_1_, PM_1-2.5_ [aerodynamic equivalent diameter >1 and ≤2.5 μm], PM_2.5_, PM_2.5-10_ [aerodynamic equivalent diameter >2.5 μm and ≤10 μm], and PM_10_ [aerodynamic equivalent diameter ≤10 μm]) prenatally and during the first year of life with childhood asthma and wheeze among children aged 3 to 6 years in China.

## Methods

### Study Population

In this cross-sectional study, from June 2019 to June 2020, a questionnaire investigation was conducted in 7 major cities in China, including Wuhan, Changsha, Taiyuan, Nanjing, Shanghai, Chongqing, and Urumqi, as the second phase of the China, Children, Homes, Health (CCHH) study.^23^ A standardized questionnaire was previously validated by a pilot study and was used in the CCHH study with written consent from the parents or legal guardians.^24^ Ethics approval for the current study was acquired from the ethical committee of the School of Public Health, Fudan University. This study followed the Strengthening the Reporting of Observational Studies in Epidemiology (STROBE) reporting guideline.

We applied a multistage cluster sampling method to choose the surveyed groups of children; the specific description of the sampling methods was provided in previous articles.^22,25^ The caregivers of children aged 3 to 6 years from 5 cities (Wuhan, Changsha, Taiyuan, Nanjing, and Shanghai) completed the standard electronic questionnaire, whereas Chongqing and Urumqi used traditional paper questionnaires to collect data. We excluded participants whose residential address was outside the survey city, children who were conceived before January 1, 2013 (the individual PM exposure was available in the 7 cities after 2013), mothers with unknown gestational time and basic covariates, and children born at less than 28 weeks’ gestation.

### Exposure Assessment

A mature machine learning–based method (an enhanced space-time extremely randomized trees model) was used to estimate the daily mean concentrations of ambient PM_1_, PM_2.5_, and PM_10_ in the 7 cities from January 2013 to December 2018, with a spatial resolution of 1 km.^26,27,28,29,30^ We further calculated the concentrations of PM_1-2.5_ and PM_2.5-10_ by subtracting the concentration of PM_1_ from PM_2.5_ and PM_2.5_ from PM_10_, respectively. The detailed descriptions of the space-time extremely randomized trees model are provided in the eMethods in the Supplement. The validation results were of high quality, with a cross-validation coefficient of determination (*CV* – *R*^2^) of 0.77 for PM_1_,^27^ 0.92 for PM_2.5_,^29^ and 0.86 for PM_10_^30^ for monthly predicted estimates, and the corresponding root mean square errors of ground measurements were 4.8 μg/m^3^, 5.1 μg/m^3^, and 11.1 μg/m^3^, respectively. We collected daily in situ measurements of PM_1_ from the China Atmosphere Watch Network and ground-based monitoring data of daily PM_2.5_ and PM_10_ from the China National Urban Air Quality Real-Time Publishing Platform from 2013 to 2018. The method of model development in this study was detailed by some of us in previous studies.^27,28,29,30^

For each participant in the present study, we first retrieved monthly mean concentrations of size-segregated particles from 2013 to 2018 from the 1 × 1-km gridded estimates based on the participant’s residential address. Monthly estimates were then used to calculate the mean exposure in early life (from the beginning of pregnancy through the first year of life) by further considering information on birth and conception dates. To reduce exposure misclassification, prenatal and first-year exposure of ambient PM was assigned based on the corresponding address information for each period.

### Respiratory Health Outcomes

The standard questionnaire, which was modified from the questionnaire of the International Study of Asthma and Allergies in Childhood,^31^ was used to collect information on lifetime-ever asthma and lifetime-ever wheeze. Participants were asked the following 2 questions: (1) “Has your child ever had doctor-diagnosed asthma?” and (2) “Has your child ever had wheezing or whistling in the chest at any time in the past?”

### Covariates

Based on the articles previously published by the CCHH study^32,33,34^ and relevant literature,^12,13,35^ we selected the following 3 groups of covariates: (1) characteristics of the child, including sex (male or female), age, ethnicity (self-identified by respondents as Han or minority nationalities and included as a covariate because this was a multicity study and there are 55 minority nationalities in China besides Han ethnicity), delivery mode (vaginal or cesarean), birth season (spring [March to May], summer [June to August], autumn [September to November], or winter [December to February]), and breastfeeding duration (<1 month, 1 month to <6 months, 6 months to <12 months, or ≥12 months); (2) characteristics of the parents, including maternal educational level (high school or below, university, or postgraduate or above), maternal smoking status (never, former, or current) and parental history of atopy (yes or no); and (3) characteristics of the household environment, including passive smoke exposure, air pollution from solid fuel, house renovation, and visible mold or dampness. The 4 household environment variables were classified as none, prenatal exposure, first-year exposure, or both.

### Statistical Analysis

Spearman correlation coefficients were calculated between pairs of size-segregated PM measures in different periods. First, multilevel (city and child) logistic regression models were applied to assess the associations of early-life (prenatal and first year) exposure to size-segregated particles with childhood asthma and wheeze. Size-segregated PM categories (PM_1_, PM_1-2.5_, PM_2.5_, PM_2.5-10_, and PM_10_) were included in the models separately. We started with the crude models (model 1); gradually added the characteristics of the child (models 2, 3, and 4), parent (models 3 and 4), and household environment (model 4); and obtained the results from the 4 models. The participants’ city was included as a random intercept in all regression models. The method of addressing missing values is provided in the eMethods in the Supplement. Based on the results of model 4, further analyses were carried out. Restricted cubic spline functions were conducted to explore the exposure-response relationships between early-life exposure to size-segregated particles and childhood asthma and wheeze. Visual inspection and a likelihood ratio test were used to examine the nonlinearity in exposure-response relationships. In addition, we further applied multilevel (city and child) logistic regression models to separately investigate the associations of prenatal and first-year exposure to size-segregated particles with childhood asthma and wheeze. Associations were calculated as odds ratios (ORs) with 95% CIs for each 10-μg/m^3^ increase in the concentration of size-segregated particles to which children were exposed. The detailed descriptions of sensitivity analyses are provided in the eMethods in the Supplement.

All statistical analyses were performed using R, version 4.0.0 (R Project for Statistical Computing). We conducted 2-sided tests and considered *P* < .05 as statistically significant.

## Results

### Characteristics of Study Population

Among 38 911 children aged 3 to 6 years, the caregivers of 37 858 (response rate, 97.3%) successfully filled out the questions for childhood asthma and wheeze; of these, we excluded 4405 children (11.6%) whose residential address was outside the survey city; 1971 (5.2%) who were conceived before January 1, 2013; 1652 (4.4%) who had a mother with unknown gestational time and basic covariates; and 412 (1.1%) who were born at less than 28 weeks’ gestation, leaving 29 418 children (77.7%) for further analyses.

Of the 29 418 included children (15 320 boys [52.1%] and 14 098 girls [47.9%]; mean [SD] age, 4.9 [0.9] years), 2524 (8.6%) were identified by their caregiver as ever having wheeze and 1161 (3.9%) were diagnosed with asthma (Table). Among all children, 15 213 (51.7%) were born vaginally, 1551 (5.3%) were preterm births, 1023 (3.5%) were low birth weight, 18 514 (62.9%) were breastfed for more than 6 months, and 787 (2.7%) had parental history of atopy. A total of 22 250 children (75.6%) had a mother with an educational level of university or above, and 576 of 25 422 children (2.3%) had a mother who was a current or former smoker during pregnancy. For household environment, 7525 of 25 422 children (29.6%) had passive cigarette smoke exposure (this information was not collected for children from Urumqi [n = 3996]). Households of 5538 of 23 548 children (23.5%) had house renovation, and households of 4440 of 23 548 (18.9%) had visible mold or dampness in the child’s early life (this information was not collected for children from Urumqi [n = 3996] or Chongqing [n = 1874]).

**Table.** Characteristics of Children, Parents, and Household Environment in the Study

Characteristic	Children (N = 29 418)^a^
Outcome	
Diagnosed with asthma	1161 (3.9)
Ever had wheeze	2524 (8.6)
Child	
Sex	
Boy	15 320 (52.1)
Girl	14 098 (47.9)
Age, mean (SD), y	4.9 (0.9)
Han ethnicity	27 882 (94.8)
Vaginal birth	15 213 (51.7)
Born in warm season (April to September)	14 834 (50.4)
Preterm birth	1551 (5.3)
Low birth weight	1023 (3.5)
Breastfeeding duration >6 mo	18 514 (62.9)
Parent	
Maternal educational level of university or above	22 250 (75.6)
Maternal smoking status (current or former)^b^	576/25 422 (2.3)
Parental history of atopy	787 (2.7)
Household environment	
Passive cigarette smoke exposure^b^	7525/25 422 (29.6)
Air pollution from solid fuel	267 (0.9)
House renovation during pregnancy or first year of life^c^	5538/23 548 (23.5)
Visible mold or dampness during pregnancy or first year of life^c^	4440/23 548 (18.9)

^a^ Data are presented as number (percentage) of children unless otherwise indicated.

^b^ Urumqi (n = 3996) did not collect information for this variable.

^c^ Urumqi (n = 3996) and Chongqing (n = 1874) did not collect information for this variable.

### Concentrations and Correlations of PM

As shown in eTable 1 in the Supplement, the mean (SD) early-life exposure to PM_1_ was 36.7 (8.9) μg/m^3^, to PM_1-2.5_ was 20.7 (4.6) μg/m^3^, to PM_2.5_ was 61.7 (13.1) μg/m^3^, to PM_2.5-10_ was 48.9 (16.6) μg/m^3^, and to PM_10_ was 110.6 (19.3) μg/m^3^. The concentrations of size-segregated particles were higher during pregnancy and then showed a decreasing trend in the first year of life, with a mean (SD) decrease of 3.9 (6.6) μg/m^3^ for PM_1_, 1.9 (6.8) μg/m^3^ for PM_1-2.5_, 7.2 (12.2) μg/m^3^ for PM_2.5_, 3.5 (10.7) μg/m^3^ for PM_2.5-10_, and 10.6 (17.5) μg/m^3^ for PM_10_. The concentrations of size-segregated particles in different periods (early life, prenatal, and first year) were highly correlated (Spearman correlation coefficient range, 0.67-0.96) (eFigure 1 in the Supplement). Moreover, the concentrations of PM_1_ and PM_2.5_ (Spearman correlation coefficient range, 0.48-0.87) and PM_2.5-10_ and PM_10_ (Spearman correlation coefficient range, 0.59-0.79) in different periods were moderately to highly correlated (eFigure 1 in the Supplement).

### Exposure to PM_1_, PM_2.5_, and PM_10_ in Early Life in Different Cities

**Figure 1.**
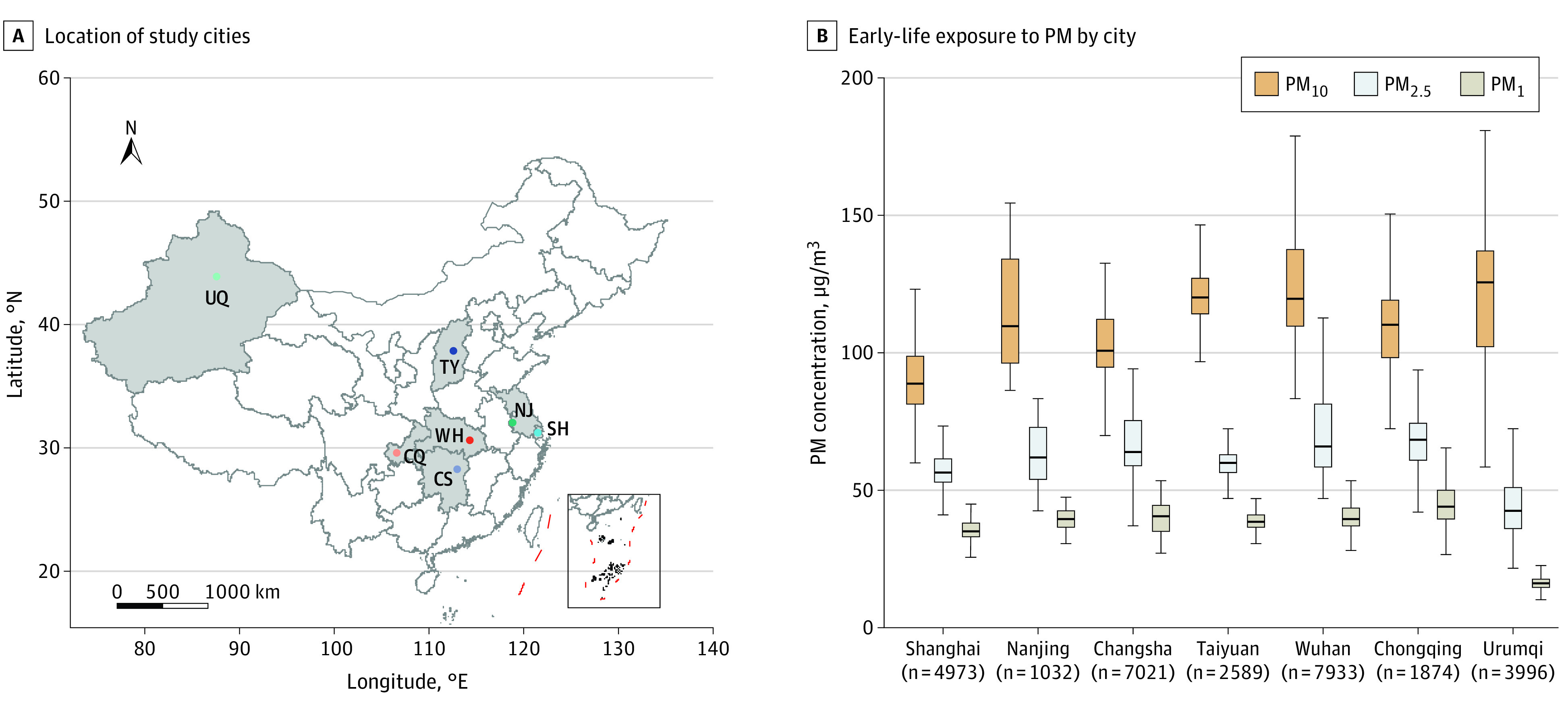
Location of Study Cities and Early-Life Exposure to Particulate Matter (PM) by City B, The horizontal line inside the boxes indicates the median PM concentration, the lower and upper ends of the boxes the lower and upper quartiles of PM concentration, and the whiskers the minimum and maximum PM concentration; subscripted numerals denote the maximum aerodynamic equivalent diameter of PM in micrometers. CS indicates Changsha; CQ, Chongqing; NJ, Nanjing; SH, Shanghai; TY, Taiyuan; UQ, Urumqi; and WH, Wuhan.

Figure 1 shows the geographic location of the study cities and concentrations of PM_1_, PM_2.5_, and PM_10_ in early life in different cities. Shanghai, a coastal city, had a median concentration of 35.0 μg/m^3^ (IQR, 32.9-37.9 μg/m^3^) for PM_1_, 56.4 μg/m^3^ (IQR, 53.0-61.1 μg/m^3^) for PM_2.5_, and 88.8 μg/m^3^ (IQR, 81.3-98.8 μg/m^3^) for PM_10_, concentrations lower than in all inland cities except Urumqi. For inland cities, the lowest median concentration of PM_10_ was found in Changsha (101.0 μg/m^3^ [IQR, 94.9-112.1 μg/m^3^]), and the lowest median concentrations of PM_1_ and PM_2.5_ were observed in Urumqi (15.8 μg/m^3^ [IQR, 14.4-17.5 μg/m^3^] and 42.3 μg/m^3^ [IQR, 35.9-51.0 μg/m^3^], respectively).

### Early-Life Size-Segregated Particles Exposure and Childhood Asthma and Wheeze

**Figure 2.**
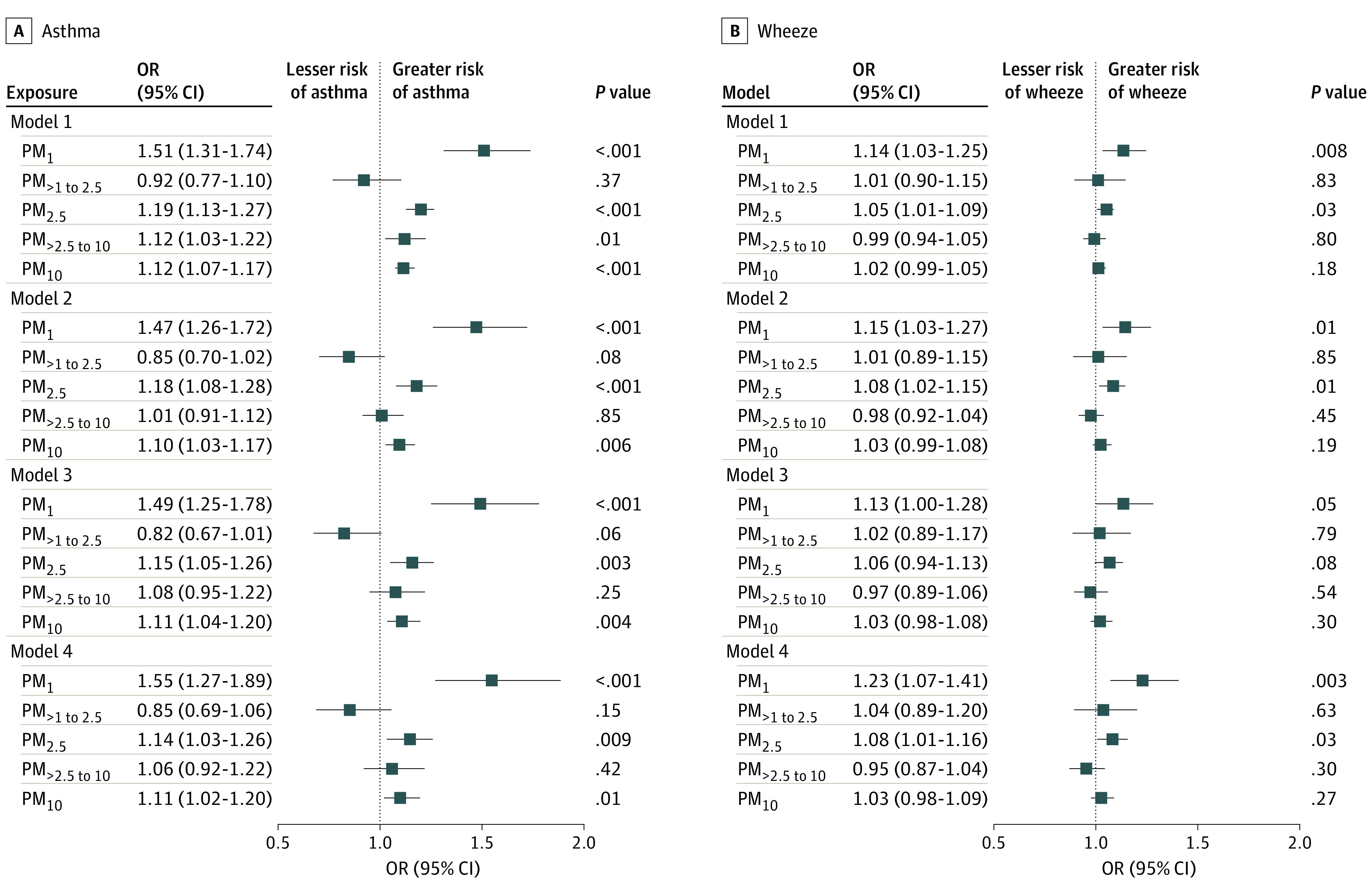
Association of Early-Life Exposure to Size-Segregated Particulate Matter (PM) With Childhood Asthma and Wheeze Model 1 was the crude model. Model 2 was adjusted for child characteristics; model 3, for child and parent characteristics; and model 4, for child and parent characteristics and household environment. Subscripted numerals denote the maximum aerodynamic equivalent diameter of PM in micrometers. Squares represent odds ratios (ORs), with horizontal lines representing 95% CIs.

Figure 2 shows the ORs and 95% CIs for the associations of early-life exposure to size-segregated particles with childhood asthma and wheeze. The estimates from model 1 (crude) to model 4 (adjusted for characteristics of children, parents, and household environments) were generally stable, indicating that the results of model 4, which included the most covariates, were robust. Each 10-μg/m^3^ increase in early-life PM_1_ and PM_2.5_ exposure was associated with an increase in the risk of childhood asthma by 55.0% (OR, 1.55; 95% CI, 1.27-1.89) and 14.0% (OR, 1.14; 95% CI, 1.03-1.26), respectively, whereas there was no association between early-life PM_1-2.5_ exposure and childhood asthma. Similarly, there was a significant association between early-life PM_10_ exposure and childhood asthma (OR, 1.11 [95% CI, 1.02-1.20] per 10-μg/m^3^ increase in PM_10_), whereas there was no association between early-life PM_2.5-10_ exposure and childhood asthma. As for childhood wheeze, we only identified associations with early-life PM_1_ exposure (OR, 1.23 [95% CI, 1.07-1.41] per 10-μg/m^3^ increase in PM_1_) and PM_2.5_ exposure (OR, 1.08 [95% CI, 1.01-1.16] per 10-μg/m^3^ increase in PM_2.5_); no associations were observed for other size-segregated particles.

**Figure 3.**
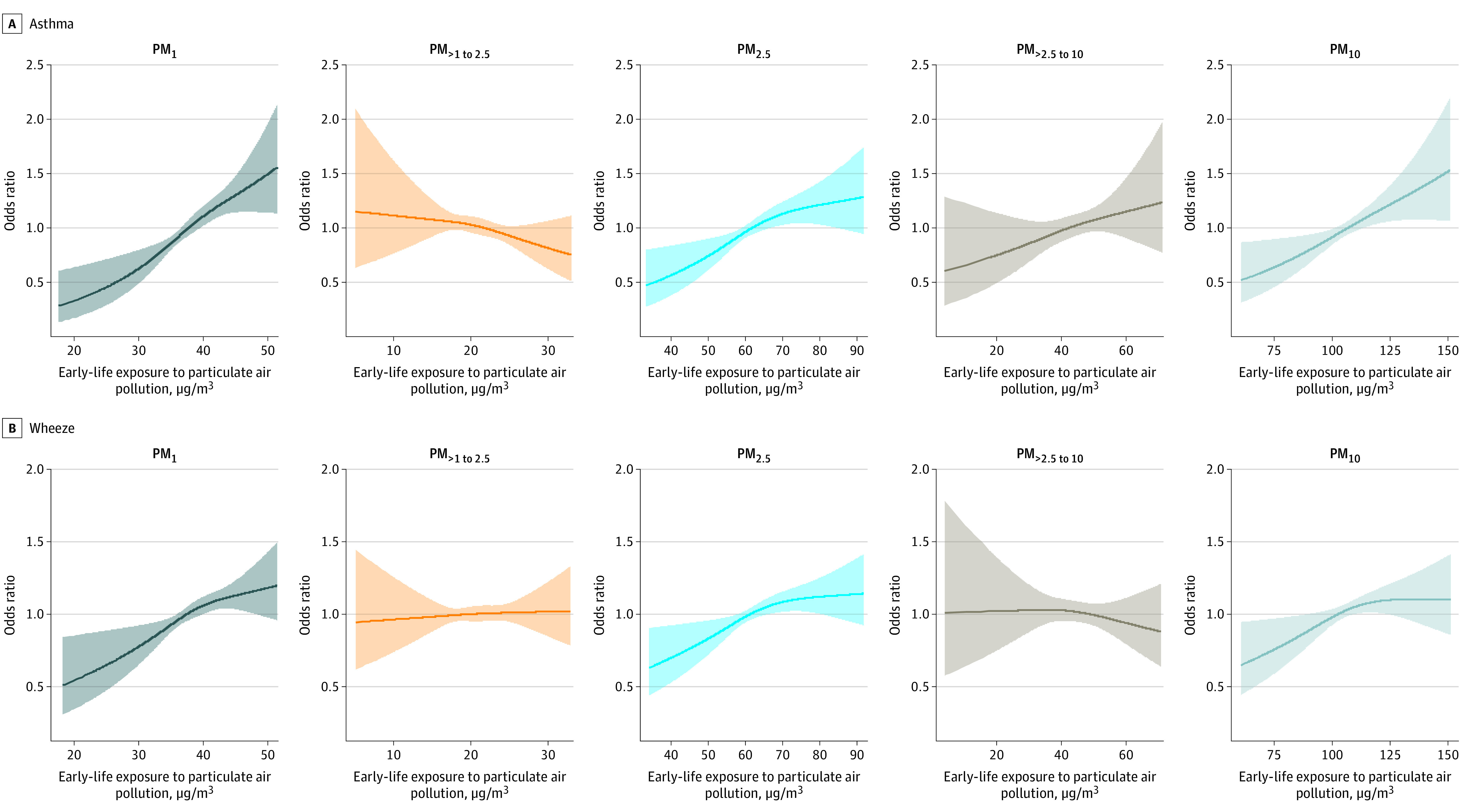
Exposure-Response Associations of Exposure to Size-Segregated Particles in Early Life With Childhood Asthma and Wheeze PM indicates particulate matter; subscripted numerals denote the maximum aerodynamic equivalent diameter of PM in micrometers. Shaded areas represent 95% CIs.

Figure 3 provides the exposure-response relationships of exposure to size-segregated particles in early life with childhood asthma and wheeze. Significant upward linear relationships of early-life PM_1_ exposure with risk of asthma and wheeze were observed. Early-life PM_2.5-10_ and PM_10_ exposure also showed upward linear relationships with the risk of asthma. Moreover, a significant nonlinear relationship of early-life PM_2.5_ exposure with risk of asthma was observed. No other exposure-response relationships were identified between size-segregated PM exposure and risk of asthma and wheeze.

### Prenatal and First-Year Size-Segregated Particles Exposure and Childhood Asthma and Wheeze

**Figure 4.**
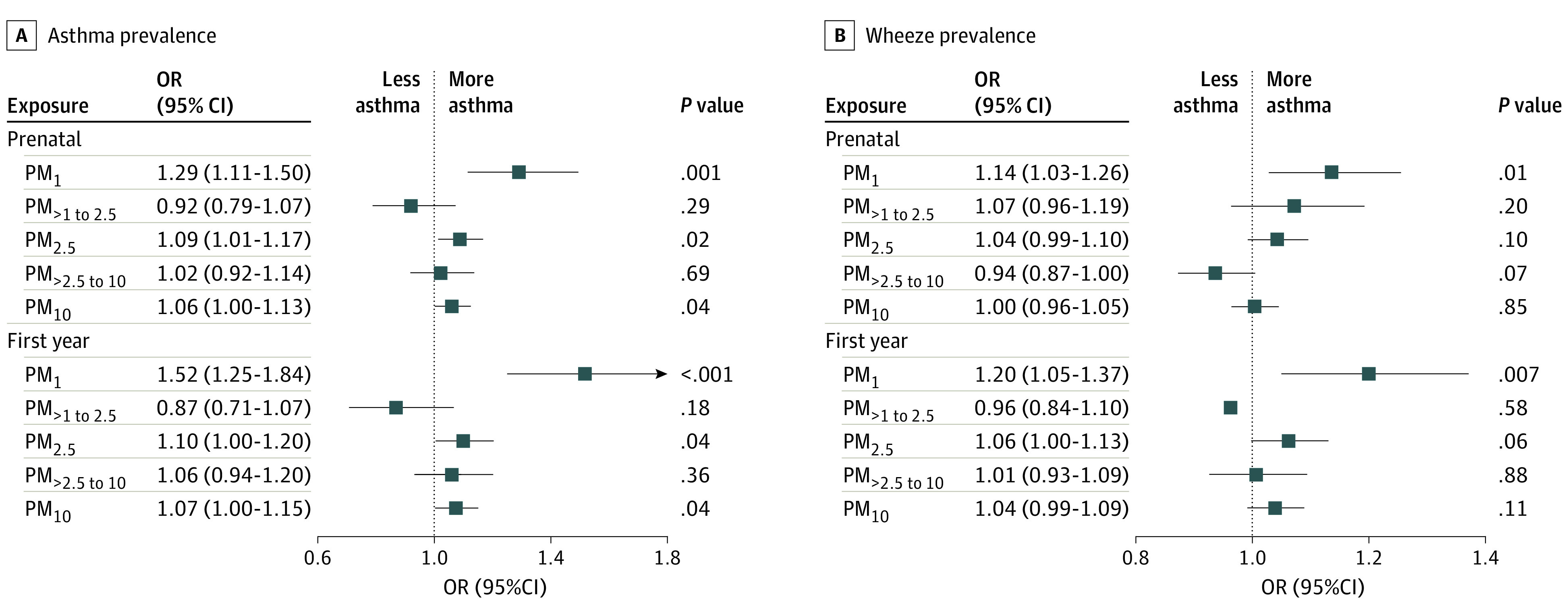
Association of Prenatal and First-Year Particulate Matter (PM) Exposure With Childhood Asthma and Wheeze Subscripted numerals denote the maximum aerodynamic equivalent diameter of PM in micrometers. Squares represent odds ratios (ORs), with horizontal lines representing 95% CIs.

As presented in Figure 4, prenatal PM_1_ exposure was positively associated with childhood asthma: for each 10-μg/m^3^ increase in exposure, the risk of childhood asthma increased by 29.0% (OR, 1.29; 95% CI, 1.12-1.50). Likewise, first-year PM_1_ exposure was associated with increased risk of childhood asthma (OR, 1.52 [95% CI, 1.25-1.84] per 10-μg/m^3^ increase in PM_1_). Compared with prenatal PM_2.5_ and PM_10_ exposure, estimates of the association of first-year PM_2.5_ and PM_10_ exposure with childhood asthma were similar (eg, OR, 1.10 [95% CI, 1.00-1.20] per 10-μg/m^3^ increase in first-year PM_2.5_; OR, 1.09 [95% CI, 1.01-1.17] per 10-μg/m^3^ increase in prenatal PM_2.5_). First-year PM_1_ exposure was associated with greater risk of childhood wheeze than was prenatal PM_1_ exposure (first-year PM_1_: OR, 1.20 [95% CI, 1.05-1.37]; prenatal PM_1_: OR, 1.14 [95% CI, 1.03-1.26]).

### Sensitivity Analyses

Similar results were found in children born at term and children overall in associations of early-life exposure to size-segregated particles with childhood asthma and wheeze (eTable 2 in the Supplement). When controlling for PM_2.5_ concentrations, the risk of childhood asthma increased by 4.0% (OR, 1.04; 95% CI, 1.02-1.05) and the risk of wheeze increased by 1.0% (OR, 1.01; 95% CI, 1.00-1.02) per 1% increase in the ratio of early-life PM_1_ to PM_2.5_ concentrations (eFigure 2 in the Supplement). No association was observed between the ratio of prenatal PM_1_ to PM_2.5_ concentrations and childhood wheeze, whereas the ratio of first-year PM_1_ to PM_2.5_ concentrations was significantly associated with childhood wheeze (OR, 1.01 [95% CI, 1.00-1.02] per 1% increase in the ratio of first-year PM_1_ to PM_2.5_ concentrations) (eFigure 2 in the Supplement).

We found that in the analyses of the associations of early-life PM_1_ exposure with childhood asthma and wheeze, the results were comparable to the original results when the concentration of PM_1-2.5_ was added to the model as a covariate (eTable 3 in the Supplement). The results of mutually adjusted analyses of associations of early-life PM_1-2.5_, PM_2.5_, and PM_2.5-10_ exposure with childhood asthma and wheeze were also found to be consistent with the original results (eTable 3 in the Supplement).

## Discussion

The results of this study indicated that early-life PM_1_, PM_2.5_, and PM_10_ exposure were associated with childhood asthma in children aged 3 to 6 years in China, with higher estimates for PM_1_ exposure. No association was observed for PM_1-2.5_, suggesting that PM_1_ rather than PM_1-2.5_ contributed to the association between PM_2.5_ and asthma. A significant upward linear exposure-response relationship was observed between early-life PM_1_ exposure and risk of asthma. In addition, significant associations between early-life PM exposure and childhood wheeze were observed for PM_1_ and PM_2.5_. To our knowledge, this is the first multicity study in China to investigate long-term associations of early-life PM_1_ exposure with childhood asthma and wheeze and to compare outcomes of PM_1_ and PM_2.5_ exposure by also assessing exposure to PM_1-2.5_.

In the present study, early-life exposure to both PM_1_ and PM_2.5_ showed consistent results regarding associations with childhood asthma and wheeze. After the exclusion of children who had an asthma diagnosis but no report of wheeze, no associations were observed between early-life PM_10_ exposure and either childhood asthma or wheeze (eTable 4 in the Supplement). The consistent results may indicate that wheeze could be one of the main factors associated with childhood asthma. Our findings are consistent with epidemiological studies of PM_2.5_ and PM_10_ exposure conducted in Canada^13,36^ and the US^37,38,39^ as well as in Shanghai,^32^ Wuhan,^22^ Changsha,^34^ and Taichung City in China.^35^ Nevertheless, discrepancies still exist across research. A study conducted in Changsha did not observe an association between prenatal PM_10_ exposure and childhood asthma^33^; a possible reason may be the difference in the exposure assessment methods because the researchers used the address of the kindergarten rather than the home address of the children. No association between PM_2.5_ exposure and childhood asthma was found in 2 studies conducted in Canada.^40,41^ The absence of an association might have been owed to the comparatively small concentration gradient of PM_2.5_, with the median concentration of PM_2.5_ in the studies being 10 μg/m^3^ and 4 μg/m^3^, respectively.

We identified statistically significant associations between early-life PM_1_ exposure and elevated risk of both childhood asthma and wheeze, showing higher estimates than for PM_2.5_ and PM_10_. Mutually adjusted PM exposure models also showed results similar to separately included PM exposure models, demonstrating the robustness of our main results. Our results were generally consistent with those of a cross-sectional study conducted in 7 cities in northeast China that revealed associations between long-term PM_1_ exposure and increased risk of asthma and wheeze and also found higher estimates with asthma.^21^ A previous single-city study conducted in Wuhan by some of us reported that prenatal rather than first-year PM_1_ exposure was associated with increased risk of childhood asthma, and neither prenatal nor first-year PM_1_ exposure was associated with childhood wheeze.^22^ The different findings between the previous study in Wuhan and the present study may be owed to the difference in the distribution of PM measures and asthma prevalence between study sites. The differences could also be attributed to the previous study^22^ estimating hazard ratios instead of ORs, as in the present study. Although the epidemiological evidence of an association between PM_1_ and respiratory diseases is limited, PM_1_ exposure in this study was associated with respiratory toxic effects. Therefore, more studies are urgently needed to explore the adverse effects of PM_1_ on human health.

In this study, we found that the association between PM_2.5_ and asthma was attributable more to PM_1_ than to PM_1-2.5_. The result of our sensitivity analyses with regard to the ratio of PM_1_ to PM_2.5_ concentrations also indicated that PM_1_ contributed to the risk of childhood asthma and wheeze associated with PM_2.5_. In a case-crossover study conducted in Shenzhen, an association with risk of hospitalization for respiratory disease was identified for short-term exposure to both PM_1_ and PM_2.5_, but not PM_1-2.5_.^42^ Likewise, data from 26 Chinese cities indicated that the association of PM_2.5_ exposure with emergency hospital visits was mostly due to PM_1_.^43^ Another study on PM-associated mortality elucidated that PM_1_ accounted for most short-term PM_2.5_-associated respiratory and chronic obstructive pulmonary disease mortality.^44^ The underlying biological mechanism may be that PM with a smaller particle size, such as PM_1_, is more likely to enter the deep respiratory tract and stimulate the alveolar wall, causing lung function impairment through oxidative stress and inflammation, and to further enter the blood circulation through the blood vessel walls.^19,45,46^ Moreover, PM_1_ has a larger active surface area, to which more toxic substances can attach.^47,48^ In a recent study in Vietnam, Hien et al reported that long-range transport aerosols, coal fly ash, and primary particulate vehicular emissions mainly appeared in PM_1_, whereas resuspended road dust and biomass-burning fly ash tended to occur in PM_1-2.5_; these findings indicate a potential underlying mechanism by which PM_1_ rather than PM_1-2.5_ contributed to the association between PM_2.5_ and asthma.^49^ These findings may be of great importance to public health for ambient PM pollution control.

### Strengths and Limitations

This study has strengths. First, an advantage of this study includes the advanced exposure assessment method using a machine learning technique. Estimates of high-resolution (1 × 1-km) spatiotemporal modeling are sufficient to accurately evaluate individual exposure. In addition, we considered a multitude of potential confounders in the adjusted models, including characteristics of children, parents, and household environments, which we believe allowed us to come to a robust conclusion.

Our study also has limitations. Due to the cross-sectional design of this study, we were unable to provide evidence of temporal and causal relationships between PM exposure and childhood respiratory outcomes. Moreover, ascertainment of childhood asthma and wheeze was acquired by self-reported questionnaires completed by caregivers and not validated by a physician; thus, they were susceptible to recall bias, and we could not determine in which direction this bias might have distorted the associations according to the present data. Most of the questionnaire respondents were mothers with university education or above, which may be due to our study selecting 7 provincial capitals in China, and this may have caused potential sampling bias. In addition, we were unable to analyze the associations of the sources and chemical composition of PM with childhood asthma and wheeze due to the lack of PM composition data. We also did not collect data on the number of wheeze episodes; thus, we were unable to further elucidate potential reasons for the inconsistent results regarding an association of PM_10_ exposure with wheeze and asthma. In addition, although educational level is a recommended and typical indicator of socioeconomic status, we did not collect any other metrics that representatively indicate socioeconomic status, such as urbanization, family income, and maternal job title; thus, we could not include other indicators of socioeconomic status as covariates in the model for analyses. Furthermore, although we included indoor air pollution from solid fuel as a covariate and outdoor PM exposure and indoor PM exposure are correlated,^50,51^ indoor PM exposure still differs among individuals. We may consider both indoor and outdoor PM concentrations as well as their chemical composition in future studies.

## Conclusions

In this study, higher estimates were observed for the association between smaller-particle PM, such as PM_1_, with childhood asthma than for PM with larger particles, suggesting that PM with a smaller particle size may be more toxic. In addition, PM_1_ was a main contributor to the association between PM_2.5_ and childhood asthma, suggesting that PM_1_ may be more important than PM with larger particles. Efforts should be continued to carry out air purification actions, effectively control PM pollution, and develop air quality guidelines for PM_1_ to reduce the adverse health impact of PMs, especially for children in China.

## Notes

Supplement.
eMethods.
**eTable 1.** Distributions of Particulate Matter Concentrations in Early Life, During Pregnancy, and in the First Year of Life **eTable 2.** Odds Ratios for the Association of Early-Life Exposure to Size-Segregated Particles With Childhood Asthma and Wheeze Among Term-Birth Children **eTable 3.** Odds Ratios for the Associations of Early-Life, Prenatal, and First-Year Exposure to Size-Specific Particles With Childhood Asthma and Wheeze When Two-Pollutant Models Were Fitted **eTable 4.** Odds Ratios for the Association of Early-Life Exposure to PM_10_ With Childhood Asthma and Wheeze After Excluding Children Who Have an Asthma Diagnosis but no Report of Wheeze **eFigure 1.** Spearman Correlation Coefficients Between Pairs of Size-Segregated PM Measures in Different Periods **eFigure 2.** Odds Ratios for the Associations of the Ratio of Early-Life, Prenatal, and First-Year PM_1_ to PM_2.5_ With Childhood Asthma and Wheeze
