# Supplementary material for: Associations of Early-Life Exposure to Submicron Particulate Matter With Childhood Asthma and Wheeze in China
Source: JAMA Netw Open. 2022 Oct 11;5(10):e2236003. doi: 10.1001/jamanetworkopen.2022.36003 (PMC9554703; doi:10.1001/jamanetworkopen.2022.36003)
Supplement: Supplement. — eMethods. eTable 1. Distributions of Particulate Matter Concentrations in Early Life, During Pregnancy, and in the First Year of Life eTable 2. Odds Ratios for the Association of Early-Life Exposure to Size-Segregated Particles With Childhood Asthma and Wheeze Among Term-Birth Children eTable 3. Odds Ratios for the Associations of Early-Life, Prenatal, and First-Year Exposure to Size-Specific Particles With Childhood Asthma and Wheeze When Two-Pollutant Models Were Fitted eTable 4. Odds Ratios for the Association of Early-Life Exposure to PM10 With Childhood Asthma and Wheeze After Excluding Children Who Have an Asthma Diagnosis but no Report of Wheeze eFigure 1. Spearman Correlation Coefficients Between Pairs of Size-Segregated PM Measures in Different Periods eFigure 2. Odds Ratios for the Associations of the Ratio of Early-Life, Prenatal, and First-Year PM1 to PM2.5 With Childhood Asthma and Wheeze [file jamanetwopen-e2236003-s001.pdf]

Supplementary Online Content

Wu C, Zhang Y, Wei J, et al. Associations of early-life exposure to submicron particulate matter with childhood asthma and wheeze in China. *JAMA Netw Open*. 2022;5(10):e2236003. doi:10.1001/jamanetworkopen.2022.36003

eMethods.
eTable 1. Distributions of Particulate Matter Concentrations in Early Life, During Pregnancy, and in the First Year of Life
eTable 2. Odds Ratios for the Association of Early-Life Exposure to Size-Segregated Particles With Childhood Asthma and Wheeze Among Term-Birth Children
eTable 3. Odds Ratios for the Associations of Early-Life, Prenatal, and First-Year Exposure to Size-Specific Particles With Childhood Asthma and Wheeze When Two-Pollutant Models Were Fitted
eTable 4. Odds Ratios for the Association of Early-Life Exposure to PM_{10} With Childhood Asthma and Wheeze After Excluding Children Who Have an Asthma Diagnosis but no Report of Wheeze
eFigure 1. Spearman Correlation Coefficients Between Pairs of Size-Segregated PM Measures in Different Periods
eFigure 2. Odds Ratios for the Associations of the Ratio of Early-Life, Prenatal, and First-Year PM_1 to $PM_{2.5}$ With Childhood Asthma and Wheeze

This supplementary material has been provided by the authors to give readers additional information about their work.

© 2022 Wu C et al. *JAMA Network Open*.

eMethods.

Space-time extremely randomized trees model

Extremely randomized trees model is one of the tree-based integrated learning methods, which divides nodes by randomly choosing cutoff points and uses all training samples to grow trees rather than the bootstrap method. Compared with other tree-based methods, variance problem can be effectively solved by this model. By introducing the spatial autocorrelation between observed values of particulate matter weighted by geographical distance (space) and temporal difference (time), the space-time randomized trees nicely integrate spatiotemporal information into extremely randomized trees model.

The space-time extremely randomized trees model can well integrated spatial and temporal data of ground measurements, aerosol optical depth retrieved by satellite, land use, topography, pollution emission, population, and meteorological information and presented good prediction performance all over mainland China.

The method of addressing missing values

Since the data of Chongqing and Urumqi were collected from paper questionnaires, some covariates were missing (as noted in the footnote of Table 1). Therefore, the data of Chongqing and/or Urumqi were not included into the analyses of the corresponding models. In other five cities using electronic questionnaires to collect data, the quality of data could be strictly controlled by setting items that could not be skipped when filling out the questionnaires to avoid the missingness of data.

© 2022 Wu C et al. *JAMA Network Open*.

Sensitivity analysis

Several studies have found relevant associations between pre-term birth and increased associated risk of asthma and other allergic diseases, while air pollution has also been associated to risk of pre-term delivery. Therefore, we further conducted sensitivity analyses only restricted to term birth children to validate the robustness of our results. Moreover, the ratio of PM_1 to $PM_{2.5}$ ($PM_1/PM_{2.5}$) can quantitatively demonstrate the contribution of PM_1 to the risk of childhood asthma and wheeze associated with $PM_{2.5}$. As sensitivity analyses, we also considered the associations of $PM_1/PM_{2.5}$ with childhood asthma and wheeze. Finally, given the impact of size-segregated particles on each other, we fitted two-pollutant models to examine the independent effect of PM_1 ($PM_{1-2.5}$) from $PM_{1-2.5}$ (PM_1) and $PM_{2.5}$ ($PM_{2.5-10}$) from $PM_{2.5-10}$ ($PM_{2.5}$) as sensitivity analyses.

© 2022 Wu C et al. *JAMA Network Open*.

eTable 1. Distributions of particulate matter concentrations in early life, during pregnancy, and in the first year of life

Exposure	Mean	SD	Percentiles				
			5th	25th	50th	75th	95th
**Early life, μg/m³**							
PM_1	36.7	8.9	15.7	33.9	37.8	42.2	48.2
$PM_{1-2.5}$	20.7	4.6	13.7	17.8	20.4	23.4	28.6
$PM_{2.5}$	61.7	13.1	38.8	54.3	60.0	69.0	85.4
$PM_{2.5-10}$	48.9	16.6	29.1	35.9	46.7	57.5	85.3
PM_{10}	110.6	19.3	79.9	96.5	109.5	123	144.7
**Prenatal, μg/m³**							
PM_1	38.9	10.5	16.2	33.9	39.3	46.1	54.1
$PM_{1-2.5}$	21.8	6.7	11.7	17.0	21.2	26.2	33.5
$PM_{2.5}$	65.8	16.2	42.3	55.0	63.0	76.1	95.5
$PM_{2.5-10}$	50.9	18.5	29.7	36.2	48.1	60.1	90.9
PM_{10}	116.6	22.9	82.4	99.4	114.2	132.4	156.6
**First year of life, μg/m³**							
PM_1	35.0	8.5	15.0	32.8	36.2	39.9	45.9
$PM_{1-2.5}$	19.9	4.8	12.8	16.8	19.6	22.4	28.6
$PM_{2.5}$	58.6	13.0	35.4	52.4	57.6	65.3	81.9
$PM_{2.5-10}$	47.5	16.5	27.1	35.1	44.7	55.3	79.3
PM_{10}	106.0	19.7	76.7	92.6	104.2	117.4	143.3

© 2022 Wu C et al. *JAMA Network Open*.

eTable 2. Odds ratios for the association of early-life exposure to size-segregated particles with childhood asthma and wheeze among term-birth children

Exposure	Asthma			Wheeze	
	OR [95% CI]	*p*-value		OR [95% CI]	*p*-value
**Model 1 [a]**					
PM_1	1.53 [1.32-1.77]	<0.001		1.13 [1.03-1.25]	0.01
$PM_{1-2.5}$	0.93 [0.77-1.13]	0.47		0.99 [0.87-1.12]	0.87
$PM_{2.5}$	1.20 [1.13-1.28]	<0.001		1.03 [0.99-1.08]	0.11
$PM_{2.5-10}$	1.12 [1.02-1.22]	0.02		0.98 [0.92-1.04]	0.47
PM_{10}	1.12 [1.08-1.18]	<0.001		1.01 [0.98-1.04]	0.52
**Model 2 [b]**					
PM_1	1.51 [1.28-1.77]	<0.001		1.15 [1.03-1.28]	0.01
$PM_{1-2.5}$	0.87 [0.71-1.06]	0.15		1.00 [0.87-1.14]	0.95
$PM_{2.5}$	1.19 [1.09-1.30]	<0.001		1.07 [1.01-1.14]	0.03
$PM_{2.5-10}$	1.00 [0.90-1.11]	0.95		0.97 [0.91-1.03]	0.36
PM_{10}	1.10 [1.03-1.18]	0.005		1.02 [0.98-1.07]	0.34
**Model 3 [c]**					
PM_1	1.54 [1.28-1.86]	<0.001		1.15 [1.01-1.30]	0.04
$PM_{1-2.5}$	0.85 [0.69-1.05]	0.13		1.00 [0.87-1.16]	0.98
$PM_{2.5}$	1.18 [1.07-1.30]	<0.001		1.06 [0.99-1.13]	0.12
$PM_{2.5-10}$	1.07 [0.94-1.22]	0.29		0.96 [0.88-1.05]	0.37
PM_{10}	1.13 [1.04-1.22]	0.002		1.02 [0.97-1.07]	0.48
**Model 4 [d]**					
PM_1	1.62 [1.31-1.99]	<0.001		1.24 [1.07-1.42]	0.003
$PM_{1-2.5}$	0.88 [0.70-1.10]	0.25		1.03 [0.88-1.20]	0.72
$PM_{2.5}$	1.16 [1.05-1.29]	0.004		1.08 [1.01-1.16]	0.04
$PM_{2.5-10}$	1.07 [0.92-1.24]	0.38		0.94 [0.86-1.04]	0.22
PM_{10}	1.12 [1.03-1.22]	0.007		1.03 [0.97-1.09]	0.35

ORs [95% CIs] were estimated for per 10-$\mu g/m^3$ increase in particulate pollution.

[a] Model 1: Crude models.

[b] Model 2: Multilevel (city and child) logistic regression models were applied and adjusted for the characteristics of child including child's sex, child's age, ethnicity, delivery mode, birth season, and breastfeeding duration.

[c] Model 3: Multilevel (city and child) logistic regression models were applied and adjusted for covariates in model 1, and characteristics of parents including maternal education level, maternal smoking status, and parental history of atopy.

[d] Model 4: Multilevel (city and child) logistic regression models were applied and adjusted for covariates in model 3, and characteristics of household environment including passive smoke exposure, air pollution from solid fuel, renovation, and visible mold/damp in early life.

© 2022 Wu C et al. *JAMA Network Open*.

eTable 3. Odds ratios for the associations of early-life, prenatal, and first-year exposure to size-specific particles with childhood asthma and wheeze when two-pollutant models were fitted

Exposure [a]		Asthma			Wheeze	
		OR [95% CI]	*p*-value		OR [95% CI]	*p*-value
**Early-life [b]**						
	PM_1	1.54 [1.27-1.89]	<0.001		1.23 [1.08-1.42]	0.003
	$PM_{1-2.5}$	0.86 [0.69-1.08]	0.19		1.03 [0.89-1.20]	0.66
	$PM_{2.5}$	1.13 [1.03-1.25]	0.01		1.08 [1.01-1.16]	0.03
	$PM_{2.5-10}$	1.05 [0.91-1.20]	0.49		0.95 [0.87-1.04]	0.25
**Prenatal [b]**						
	PM_1	1.30 [1.12-1.51]	<0.001		1.13 [1.02-1.25]	0.02
	$PM_{1-2.5}$	0.90 [0.77-1.05]	0.17		1.05 [0.95-1.17]	0.35
	$PM_{2.5}$	1.08 [1.01-1.17]	0.03		1.04 [0.99-1.10]	0.10
	$PM_{2.5-10}$	1.02 [0.92-1.13]	0.77		0.94 [0.87-1.00]	0.06
**First-year [b]**						
	PM_1	1.53 [1.25-1.86]	<0.001		1.21 [1.06-1.38]	0.006
	$PM_{1-2.5}$	0.90 [0.73-1.11]	0.33		0.96 [0.84-1.10]	0.58
	$PM_{2.5}$	1.09 [1.00-1.20]	0.06		1.06 [1.00-1.13]	0.05
	$PM_{2.5-10}$	1.04 [0.92-1.18]	0.53		0.99 [0.92-1.08]	0.89

ORs [95% CIs] were estimated for per 10-μg/m^3 increase in particulate pollution.

[a] Adjusted for $PM_{1-2.5}$ in PM_1 models, adjusted for PM_1 in $PM_{1-2.5}$ models, adjusted for $PM_{2.5-10}$ in $PM_{2.5}$ models, and adjusted for $PM_{2.5}$ in $PM_{2.5-10}$ models.

[b] Multilevel (city and child) logistic regression models were applied and adjusted for child's sex, child's age, ethnicity, delivery mode, birth season, breastfeeding duration, maternal education level, maternal smoking status, parental history of atopy, passive smoke exposure, air pollution from solid fuel, house renovation, and visible mold/damp in early life.

© 2022 Wu C et al. *JAMA Network Open*.

eTable 4. Odds ratios for the association of early-life exposure to PM_{10} with childhood asthma and wheeze after excluding children who have an asthma diagnosis but no report of wheeze

PM_{10} exposure	Asthma			Wheeze	
	**OR [95% CI]**	***p*-value**		**OR [95% CI]**	***p*-value**
Model 1 [a]	1.11 [1.05-1.17]	<0.001		1.02 [0.99-1.05]	0.14
Model 2 [b]	1.06 [0.98-1.16]	0.14		1.03 [0.99-1.08]	0.15
Model 3 [c]	1.08 [0.99-1.18]	0.09		1.03 [0.98-1.09]	0.24
Model 4 [d]	1.08 [0.98-1.19]	0.13		1.03 [0.98-1.09]	0.23

ORs [95% CIs] were estimated for per 10-$\mu g/m^3$ increase in particulate pollution.

[a] Model 1: Crude models.

[b] Model 2: Multilevel (city and child) logistic regression models were applied and adjusted for the characteristics of child including child's sex, child's age, ethnicity, delivery mode, birth season, and breastfeeding duration.

[c] Model 3: Multilevel (city and child) logistic regression models were applied and adjusted for covariates in model 1, and characteristics of parents including maternal education level, maternal smoking status, and parental history of atopy.

[d] Model 4: Multilevel (city and child) logistic regression models were applied and adjusted for covariates in model 3, and characteristics of household environment including passive smoke exposure, air pollution from solid fuel, renovation, and visible mold/damp in early life.

© 2022 Wu C et al. *JAMA Network Open*.

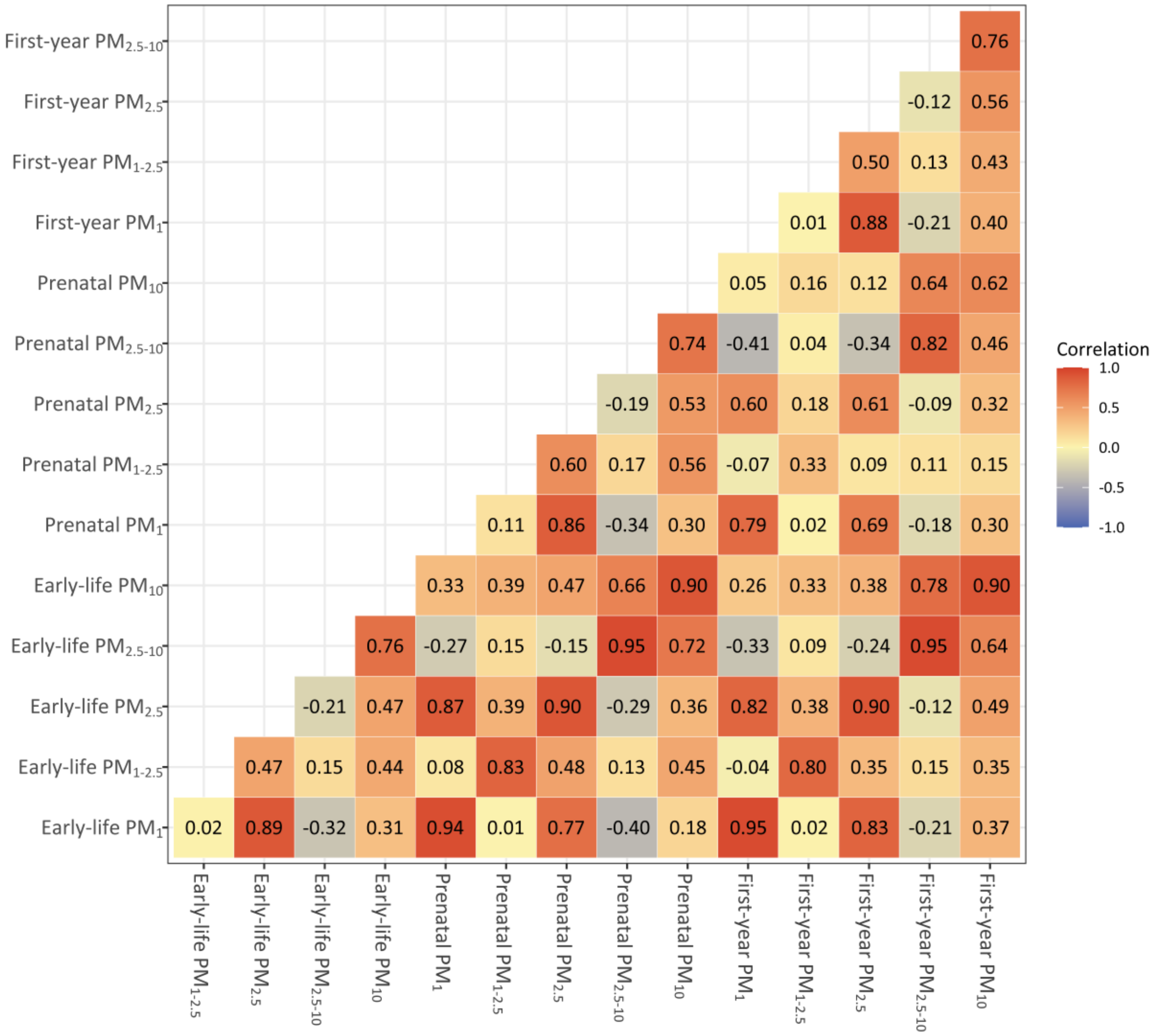

eFigure 1. Spearman correlation coefficients between pairs of size-segregated PM measures in different periods

© 2022 Wu C et al. *JAMA Network Open*.

Exposure	Asthma Prevalence OR [95% CI]	Asthma Prevalence *P*-value	Wheeze Prevalence OR [95% CI]	Wheeze Prevalence *P*-value
**Early-life**				
Model 1	1.029 [1.015-1.044]	<0.001	1.008 [0.999-1.017]	0.084
Model 2	1.028 [1.014-1.042]	<0.001	1.008 [0.999-1.017]	0.097
Model 3	1.034 [1.017-1.050]	<0.001	1.007 [0.996-1.018]	0.202
Model 4	1.035 [1.017-1.054]	<0.001	1.011 [0.999-1.023]	0.079
**Prenatal**				
Model 1	1.021 [1.010-1.032]	<0.001	1.003 [0.996-1.011]	0.371
Model 2	1.019 [1.008-1.031]	<0.001	1.004 [0.996-1.011]	0.336
Model 3	1.020 [1.007-1.033]	0.002	1.002 [0.993-1.011]	0.672
Model 4	1.024 [1.010-1.039]	<0.001	1.005 [0.995-1.015]	0.312
**First-year**				
Model 1	1.025 [1.012-1.038]	<0.001	1.010 [1.002-1.019]	0.017
Model 2	1.025 [1.013-1.038]	<0.001	1.010 [1.002-1.018]	0.019
Model 3	1.030 [1.016-1.045]	<0.001	1.010 [1.000-1.020]	0.056
Model 4	1.030 [1.014-1.047]	<0.001	1.013 [1.002-1.024]	0.025

Axis: 0.97 1.00 1.03 1.06 (both panels)

eFigure 2. Odds ratios for the associations of the ratio of early-life, prenatal, and first-year PM_1 to $PM_{2.5}$ with childhood asthma and wheeze. ORs [95% CIs] were estimated for per 1% increase in the ratio of PM_1 to $PM_{2.5}$. Model 1: Crude models, only adjusted for the concentration of $PM_{2.5}$. Model 2: Multilevel (city and child) logistic regression models were applied and adjusted for the concentration of $PM_{2.5}$ and the characteristics of child including child's sex, child's age, ethnicity, delivery mode, birth season, and breastfeeding duration. Model 3: Multilevel (city and child) logistic regression models were applied and adjusted for the concentration of $PM_{2.5}$, covariates in model 2, and characteristics of parents including maternal education level, maternal smoking status, and parental history of atopy. Model 4: Multilevel (city and child) logistic regression models were applied and adjusted for the concentration of $PM_{2.5}$, covariates in model 3, and characteristics of household environment including passive smoke exposure, air pollution from solid fuel, renovation, and visible mold/damp in early life.

© 2022 Wu C et al. *JAMA Network Open*.
